# Surface Oxidation of Nano-Silicon as a Method for Cycle Life Enhancement of Li-ion Active Materials

**DOI:** 10.3390/molecules25184093

**Published:** 2020-09-07

**Authors:** Maciej Ratynski, Bartosz Hamankiewicz, Dominika A. Buchberger, Andrzej Czerwinski

**Affiliations:** Faculty of Chemistry, University of Warsaw, Pasteura 1, 02-093 Warsaw, Poland; mratynski@chem.uw.edu.pl (M.R.); daziolkowska.edu@gmail.com (D.A.B.); aczerw@chem.uw.edu.pl (A.C.)

**Keywords:** Li-ion, silicon, oxidation, silicon oxide, cycle life

## Abstract

Among the many studied Li-ion active materials, silicon presents the highest specific capacity, however it suffers from a great volume change during lithiation. In this work, we present two methods for the chemical modification of silicon nanoparticles. Both methods change the materials’ electrochemical characteristics. The combined XPS and SEM results show that the properties of the generated silicon oxide layer depend on the modification procedure employed. Electrochemical characterization reveals that the formed oxide layers show different susceptibility to electro-reduction during the first lithiation. The single step oxidation procedure resulted in a thin and very stable oxide that acts as an artificial SEI layer during electrode operation. The removal of the native oxide prior to further reactions resulted in a very thick oxide layer formation. The created oxide layers (both thin and thick) greatly suppress the effect of silicon volume changes, which significantly reduces electrode degradation during cycling. Both modification techniques are relatively straightforward and scalable to an industrial level. The proposed modified materials reveal great applicability prospects in next generation Li-ion batteries due to their high specific capacity and remarkable cycling stability.

## 1. Introduction

The majority of currently produced Li-ion cells contain layered transition metal oxides as cathodes [1] and graphite or semi-graphitized carbon as anode active materials [2,3]. New active materials are under intensive development in terms of their capacity, power density or cycle life enhancement. One of the most promising, next generation negative electrode active materials are Group IV elements such as silicon, germanium, tin, and lead [4,5,6,7], as well as their corresponding oxides or nitrides [8,9,10]. In this group, the silicon presents the highest specific capacity (3590 mAh g^−1^), which is almost ten times greater than presently used graphite’s (372 mAh g^−1^) [11], and over three times higher than tin’s (993 mAh g^−1^) [5]. Germanium and lead draw much less attention due to their high price, weight or environmental toxicity. The high specific capacity of silicon is a tremendous advantage compared to currently used materials, however during the lithiation, formation of Li-Si alloy can lead to material expansion by up to 300% resulting in a fast mechanical degradation of the electrode layer or/and fast solid electrolyte interface (SEI) layer formation and internal resistance buildup [12]. Despite the high total volume change during lithiation, silicon is characterized by the lowest relative volume change per capacity unit (0.08% g (mAh)^−1^), lowest price and highest abundance among all Group IV elements (except from carbon). Multiple theoretical and experimental studies had been done towards stabilization of the silicon electrodes cycling performance. Many theoretical papers focus on a stress generation modeling [13,14,15] and have led to a better understanding of the mechanical processes that cause particle pulverization and a delamination from the active mass. Recent models [13,15] and experiments [16,17] predict a large stress formation during the beginning of the silicon lithiation, and even greater stress generation when switching the current direction. Based on those models, the cycle life improvement of the Si-based electrodes can be done via operation at a high lithiation level and by minimizing partial discharge cycles.

The experimental investigations mostly focus on the silicon nanostructure development. It has been proven that small silicon grains, typically below 150–300 nm (this depends on the shape) are very unlikely to crack during lithiation [18]. Amorphous silicon is less prone to cracking due to an anisotropic volume increase. Amorphous silicon nanoparticles (NPs) can remain crack-resistant when their diameter is below 870 nm [19,20]. The most promising papers describing the electrode development by the silicon nanostructure formation present the nano Si/C stack multilayer prepared by the chemical vapor deposition (CVD) method [21], carbon hollow spheres with silicon NPs [22], silicon hollow spheres with carbon coating made through sacrificial core dissolution [23], silicon NPs embedded into graphite 3D matrix [24], silicon nano-pillars, nano-wires or self-sustainable porous silicon structures [25,26,27], composites with graphite [28] or lithium titanium oxide [29] and graphene layers [30]. Nanostructures containing empty space (e.g., pillars, wires) are beneficial for the silicon cycle life, because they provide the space for an unrestricted volume change without electrode macro-structure degradation. The elastic amorphous carbon matrix, which can buffer the silicon volume changes, provide similar but less promising effects. In general, the discussed nanostructure modification methods offer the electrodes with a high initial capacity and cycle stability. However, these electrodes also reveal a high initial irreversible (IRR) capacity (1000–2500 mAh g^−1^ of IRR) related to the electrolyte decomposition and SEI formation due to the very high electrochemically active surface area (ECSA) [31]. Most of the nano-structure generation techniques are also time consuming and difficult to scale up to industrial levels. Composites of silicon with other active materials prepared by a standard slurry method commonly result in a lower SEI formation charge, but also in a lower initial capacity (due to their much lower Si content) and a poor cycle life. A typical composite capacity retention is about 65–75% after 100 cycles [29,32].

Silicon oxides (SiO_x_) or nitrides (SiN_x_) have gained more attention due to a possible diminishing of the volume expansion during material lithiation [33,34]. During the first lithiation, these materials should transform into elemental silicon and lithium oxide/nitride, respectively. The large amount of inactive compounds incorporated in the active mass act as a volume buffer and result in an improvement of the electrode cycle life. Silicon nitride often forms Li_2_SiN_2_ [33], however the observed cycle stability of the cell is only slightly enhanced [9]. Much better effects can be achieved by using a non-stoichiometric silicon oxide. SiO_x_-based electrodes present a much higher capacity retention compared to silicon-nitride-based ones. Utilization of SiO_1.1_ resulted in over 90% capacity retention after 25 cycles, while a lower oxygen content (SiO_0.8_) electrode, prepared under the same conditions, retained less than 50% of its initial capacity [34]. Formation of lithium oxide limits the Si NPs’ agglomeration effectively and reduces the electrode degradation due to silicon swelling/shrinking. The main disadvantage of using SiO_x_ compounds is their very high irreversible capacity and lithium loss related to Li_2_O formation. The first cycle efficiency can be lower than 50%, which is unacceptable in a commercial application. Other drawbacks are a much smaller specific capacity of SiO_x_ electrodes (typically less than 1000 mAh g^−1^), compared to pure silicon [8,35], and much higher internal resistance due to a poor electrical conductivity of the generated lithium oxide.

A recent study proved that lithium silicates, with different stoichiometry, are formed during the lithiation of the native SiO_2_ layer, naturally present at the silicon surface [36]. The actual composition of these silicates depends on the initial oxide characteristics and the electrode potential. Some of the formed compounds (for example Li_4_SiO_4_) are capable of efficient lithium ion transport. As a result, the cell power capabilities are expected to improve. The partial lithiation of the SiO_2_ into Li_2_Si_2_O_5_ can be fully reversible. The discussed effect was observed during biosilica lithiation at 0.9 V vs. Li [35]. Despite these promising results, in most cases, silicon oxide-based electrodes present a lower specific capacity and power, as well as a high IRR during the first cycle, thus their application in commercial batteries is still hindered.

Here we present chemical methods for silicon NP modifications resulting in partial surface oxidation. The modifications result in formation of a silicon oxides layer at the Si-core surface. The different thickness and porosity of the oxides obtained through two separate methods change the material electrochemical properties. The obtained materials combine high silicon specific capacity along with the enhanced capacity retention of SiO_x_ compounds. The presented methods are straightforward and easily scalable, and thus might be a great improvement for next generation battery technology.

## 2. Materials and Methods

Silicon nano-powder (#795585, Sigma Aldrich, Saint Louis, MO, USA) was modified by partial oxidation using two different chemical procedures. The pristine NPs of a diameter less than 100 nm have a natural oxide content below 3%.

The first procedure involved a pristine Si powder chemical oxidation in a H_2_O_2_/H_2_SO_4_ (piranha) solution at elevated temperature. The pristine powder was mixed with the piranha solution: 1 part of 30% H_2_O_2_ and 3 parts of 95% H_2_SO_4_ (*v*/*v*) at room temperature. The mixture was then heated to 70 °C and stirred for 1 h. Next it was cooled down to room temperature. The modified powder (Si-1) was separated from the solution by centrifugation, washed four times with distilled water (separated by centrifugation each time) and dried in a vacuum oven at 80 °C.

The second procedure involved: (1) a chemical dissolution of the native oxide layer by hydrofluoric acid (HF) generated in-situ, followed by (2) an oxidation step used in the first procedure. The HF-induced dissolution of native silicon oxide was performed for 30 min in 40% NH_4_F, 35% HCl and water solution (5:1:10, *v*/*v*) at room temperature. Afterwards, the product (Si-2) was separated from the solution by centrifugation and immediately transferred into the piranha solution. The subsequent steps were identical to those of the first procedure.

The effects of surface modifications were evaluated by X-ray photoelectron spectroscopy (XPS). performed using an Axis Supra instrument (Kratos, Manchester, UK) equipped with an Al Kα monochromatic beam (1486.7 eV) as X-ray source. The X-ray takeoff angle was 45°. The pass energies were set to 160 eV and 20 eV for low- and high-resolution spectra acquisitions, respectively. The data treatment was performed in the CasaXPS software. All spectra were calibrated by using the C 1s adventitious carbon line as a reference binding energy (284.8 eV).

Obtained powders were mixed with XC-72 conductive carbon (Cabot, Boston, MA, USA) and 4% water solution of carboxymethylcellulose (CMC) in a 6:2.5:1.5 weight ratio. The formed slurry was deposited on a copper foil and dried at 80 °C in the vacuum oven. All cells were assembled using a 3-electrode casing (Swagelok^®^, Solon, OH, USA). A counter electrode and a reference electrode were composed of metallic lithium (Sigma Aldrich). The electrolyte contained 1M LiPF_6_ in 3:7 fluoroethyl carbonate (FEC): ethyl methyl carbonate (EMC) + 2% vinylidene carbonate (VC).

The electrochemical testing was performed using a 1287 potentiostat/gavanostat (Solartron, Hampshire, UK)—CV experiments and a 0961 battery cycler (Atlas-Sollich, Rebiechowo, Poland)—galvanostatic cycling. Before the testing, all cells were kept for 24 h at OCV to achieve internal equilibrium. Charge/discharge cycling tests performed at 0.1 C between 1.6–0.02 V vs. Li/Li^+^. The cycle voltammetry (CV) was conducted with a scan rate of 0.2 mV/s. A FE-SEM Merlin by Carl Zeiss (Oberkochen, Germany) with an EDS analyzer was used to obtain scanning electron microscope images and elemental compositions.

## 3. Results and Discussion

### 3.1. X-ray Photoelectron Spectroscopy

**Figure 1:**
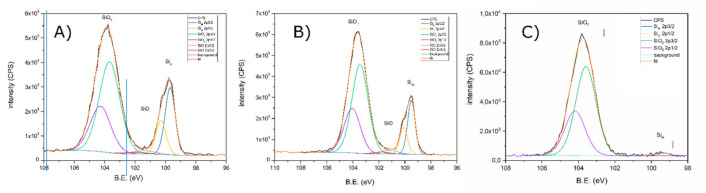
Si 2p band revealed by high-resolution XPS measurement for pristine powder (**A**), after modification 1 (**B**), and modification 2 (**C**).

The XPS analysis showed that the pristine powder surface is composed mostly of oxygen-, sulfur- and nitrogen-containing compounds (Table 1). The main difference between the three investigated powders was related to a different elemental silicon to silicon oxides ratio (Figure 1). The XPS analysis of modified samples revealed a lower elemental silicon content (21.4 and 2.1% for Si-1 and Si-2 samples, respectively). This variation is the effect of the different silicon oxide layer thickness at the grain surface. The photoelectron effective signal acquisition depth is in the range of 5–15 nm. The detected elemental silicon signal originates from the silicon core, which is present under the top oxide layer. The reduced Si_el_ signal among modified powders indicate a much thicker oxide coating on those materials. The Si-2 surface oxide layer is thick enough to almost completely block the Si_el_ signal, indicating the surface oxide layer thickness in the range of photoelectron penetration (~15 nm). The Si-1 powder analysis showed that the Si_el_ signal was only 5% smaller compared to the pristine powder, thus the very minor oxide thickness buildup occurred during first procedure. It can be also observed that reduction of the native oxide layer during procedure 2, resulted in an absence of SiO in the final product (Si-2).

A large difference in the oxide thickness between Si-1 and Si-2 powders may originate from two different mechanisms: (a) The native oxide layer is so dense and compact, that it almost completely blocks the access of the oxidizing solution to the elemental silicon core, and as a consequence, inhibits the reaction; (b) The traces of HF transferred to the piranha solution during the second procedure lead to silicon oxide dissolution. In that situation the dissolution of SiO_2_ reveals a fresh Si surface, which is immediately oxidized by the piranha solution. The process continues until all the HF is consumed. The dissolution/oxidation process may proceed with different kinetics at different silicon crystal planes or due to local substrate concentration differences. In that situation, the oxidation/dissolution processes will generate porous oxide structure embedded deeply in the silicon core. As a result of this two opposite reactions a thick oxide layer will be formed in a process similar to Al or Ti oxidation in HF solution [37,38].

The small differences in chemical shift of various forms of silicon-oxygen bonds makes it very difficult to separate the signals from individual compounds/surface groups. The possible determination of small differences in the silicon oxide morphology obtained by modification 1 and 2 will be the scope of further studies.

A small signal (2.7 at.%) related to the presence of nitrogen compounds was found at 402.95 eV, which is the most likely a result of sample contamination during Si NP production. Nevertheless, there was no N KLL line visible in the wide scan, which should be detected even when a small amount of nitrogen is present in the sample. The chemical shift of N 1s (402.95 eV) is close to the region of nitrogen in silicone oxynitride [39]. Unfortunately, this chemical shift is also in good agreement with other nitrogen compounds bonded with oxygen, e.g., nitrates, nitrated organic compounds, etc. As a result, the direct attribution of the observed signal to a specific nitrogen form was not possible. The overall nitrogen quantity in the modified samples was much lower compared to the untreated material, indicating that the observed compound, whatever its nature, was partially washed away during chemical treatment.

The sulfur contamination originated from the presence of SO_4_ groups. The magnitude of the sulfur line in the Si-1 sample was slightly higher compared to pristine material due to the chemical modification procedure involving the use of H_2_SO_4_. Interestingly the Si-2 sample presented a lower sulfur content.

The Si-2 powder surface was covered with fluorine compounds related to HF utilization in the first part of the modification procedure. The fluorine signal presence originated from Si-F and C-F compounds—probably from partially fluorinated silicon and hydrocarbon compounds or adsorbed SiF_6_^2−^ anions formed during SiO_2_ dissolution.

### 3.2. SEM Microscopy

**Figure 2:**
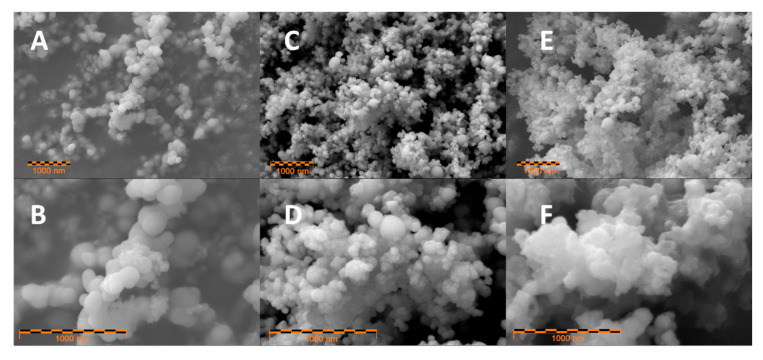
SEM images of pristine Si NPs (**A**,**B**), Si-1 (**C**,**D**) and Si-2 (**E**,**F**) samples. Upper figures—magnification 20,000×, lower—50,000×.

SEM imaging revealed that both of the modified samples contained small nanoparticles with an average diameter of 100 nm (Figure 2C–F). The main difference between the Si-01 and Si-02 samples can be observed at higher magnifications. The sample modified with piranha solution (Si-01) has well separated NPs (similar to a pristine material—Figure 2A), while the sample modified with hydrofluoric acid and piranha solution (Si-02) is mostly composed of pristine silicon NPs encapsulated in a dense, agglomerated matrix. This well-visible matrix is additional evidence of the thick silicon oxide layer growth on top of the previously HF-etched Si NPs. The in-situ silicon oxide layers probably interconnect and form a shared oxide matrix that acts as a “glue”. Consequently, the silicon particles are encapsulated in the silicon oxide matrix and thus large, uniform core/shell structures are formed.

It can be assumed that the agglomerated oxide matrix is strongly connected to the Si core and does not undergo separation during slurry preparation, leading to the agglomerated structure presence in the final electrode layer. The effect of agglomeration on the electrochemical behavior may be dual. Larger structures may lead to less uniform mixing with conductive carbon and binder in the slurry. On the other hand, oxide matrix may stabilize the silicon NPs during volume changes. The impact of different oxides structure on the electrode parameters was further evaluated by a series of electrochemical experiments.

The EDX analysis revealed gradual changes of the oxygen content in modified samples. In good accordance with the XPS analysis, the EDX data showed that the Si-1 sample has the oxide content very similar to that of pristine NPs. The slight increase in the oxygen content is related to surface oxidation by piranha solution. The Si-2 sample results showed a major increase in the oxygen content related to the thick oxide layer formation after the modification procedure. The O/Si ratio for Si-2 sample is over two times higher than for pristine NPs and Si-1 material. The ratio, however, is lower that 2.0 (expected for pure SiO_2_ phase) indicating the presence of elemental silicon underneath the surface oxide layer. The exact content of elemental silicon is hard to determine due to the presence of SiO_x_, however the estimated oxide thickness (initial particle radius = 50 nm) is close to 6.6, 8.2 and 17.8 nm for the pristine, Si-1 and Si-2 samples, respectively. The estimated oxide thickness is in good agreement with XPS data that revealed very low amount of elemental silicon content in the Si-2 sample. The XPS maximum excitation depth is close to 15 nm. The oxide thickness of Si-2 sample is higher, leading to a lack of Si signal in the XPS analysis.

The EDX analysis revealed small amounts of sulphur (Si-1 and Si-2) and fluorine (only Si-2) contamination due to the presence of residues remaining after the modification procedures. The amount of contaminants was significantly higher in the Si-2 sample (S at. % = 1.33, F at. % = 2.07) compared to the Si-1 sample (S at. % = 0.58). This was probably related to an adsorption of Sulphur- and fluorine-containing species in the thick and porous oxide layer. The comparison with XPS results revealed a much lower sulphur content in the EDX analysis, leading to the conclusion that S-containing residues are present mostly on the surface of the oxide layer. The fluorine concentration was similar in both experiments (XPS and EDX) which suggests a uniform distribution of F-containing species in the whole oxide layer. Detailed results obtained by the EDX analysis can be found in Table 2 and the Supplementary Information (Figures S3 and S4).

**Figure 3:**
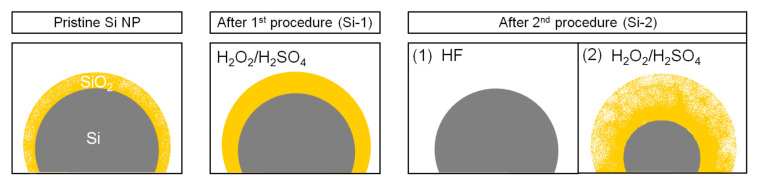
Schematic representation of the effect of performed procedures to the Si-oxide layer.

Based on XPS and SEM analyses it can be assumed, that two materials produced by different procedures are characterized by distinct morphology. The grains of Si-1 sample (one-step preparation) are covered by thin, dense layer of silicon oxide, while Si-2 sample (two-step procedure) is distinguished by thick porous coating (Figure 3).

### 3.3. Electrochemical Results

**Figure 4:**
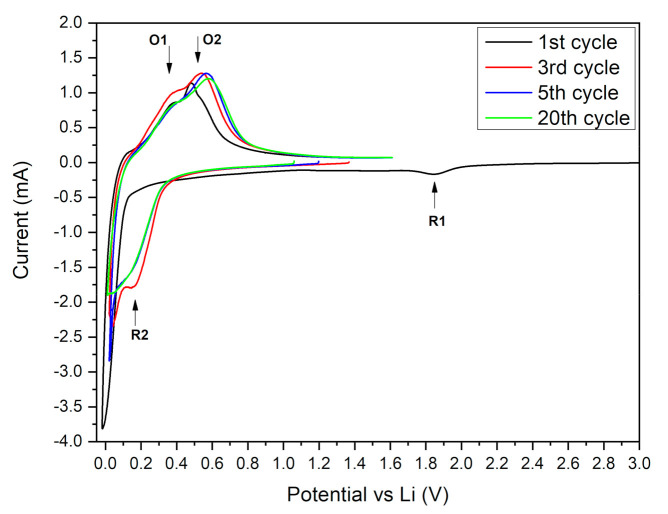
Cycle voltammetry of Si-1 electrode in cycle 1, 3, 5 and 20.

Cyclic voltammetry (CV) was performed at a 0.2 mV/s sweep rate. The measurements were conducted before the 1st (freshly assembled), and after 3rd, 5th and 20th galvanostatic cycle. The results show big differences between the CV profiles of the modified powders. During the 1st scan of the Si-1 sample (thin oxide layer), the cathodic part contains only one very small peak at 1.8 V vs. Li/Li^+^ (R1 at Figure 4). This peak is probably related to a partial reduction of the surface oxide to lithium silicate. No obvious peaks at the lower potential range (1.7–0.3 V vs. Li/Li^+^) were detected indicating a very small SEI generation charge. The further lithium silicate reduction to Li_x_SiO_y_ and subsequent reduction to Li_2_O and Si_el_ should proceed at 1.3 V and 0.7 V as stated in the literature [36,40,41]. The lack of these peaks indicates that the generated lithium silicate layer is somehow resistant to electro-reduction. The very dense and compact structure of the formed oxide might be the cause of this phenomenon. Silicon oxide is a very poor electronic conductor and restrains the electrolyte decomposition. The formed dense oxide acts as artificial SEI layer, seals the electrode surface and leads to a smaller electrolyte components’ reduction charge during the first cathodic scan. During the first scan, the cathodic part contains a sharp peak starting at ca. 0.2 V vs. Li/Li^+^ related to the silicon two-phase lithiation process. In further scans, the mechanism of silicon lithiation changes to two separate one-phase reactions reflected by the formation of a R2 peak at higher potential (Figure 4). During an oxidation scan of the Si-1 sample, two peaks: O1 and O2 (Figure 4) are clearly visible.

These peaks correspond to well-known reactions of the Li-Si alloy delithiation, proceeding through a Li_7_Si_12_ intermediate phase. The reaction related to peak O1 is much slower compared to the subsequent intermediate phase delithiation. The O1 peak is highly broadened, shifted towards positive potentials, and for that reason highly imposed onto the O2 peak. The Li-Si alloy oxidation mechanism is similar during the first 20 cycles, however a small shift towards a positive potential value can be seen between cycle 1 and 3 indicating a decrease of the reaction kinetics during the 3rd and following cycles. The O2 peak potential shift between 5th and 20th cycle of the Si-1 is almost indistinguishable, suggesting a high electrochemical and mechanical stability of Si-1 electrodes.

**Figure 5:**
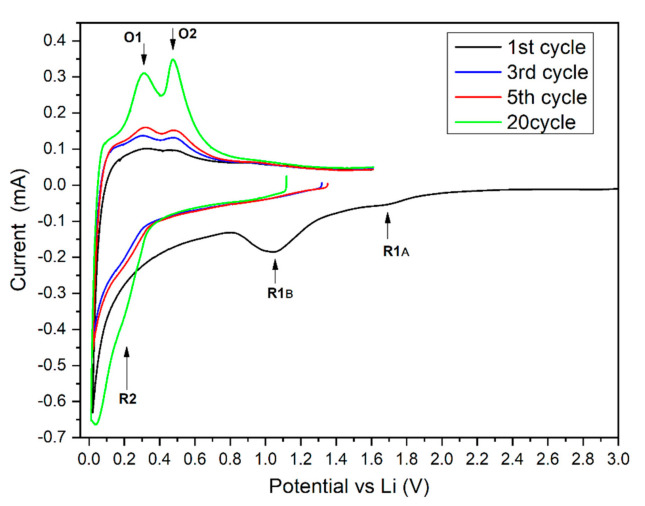
Cycle voltammetry of Si-2 electrode in cycles 1, 3, 5 and 20.

The CV analysis of the Si-2 sample revealed a completely different electrochemical behavior. During the first scan, two peaks related to a silicon oxide reduction to lithium silicates are visible (R1A and R1B). The large area of the R1B peak (Figure 5) indicates that a high percentage of lithium silicate was reduced to Li_x_SiO_y_. At potentials between 0.3–0.8 V, no obvious peak is visible, however a high cathodic current flow suggests the high electrolyte decomposition and SEI formation rate. The lack of the peak related to the SEI formation may be related to a poor conductivity of the reduced lithium silicate that kinetically hinders the reaction and leads to the severe SEI formation peak broadening. The appearance of R2 peak during the 3rd and further cycles indicates the silicon lithiation through the mechanism similar to that observed for sample Si-1. The oxidation (delithiation) of the Si-2 material proceeds through the intermediate phase formation similar to the Si-1 electrode. The separation of O1 and O2 peaks (Figure 5) is much greater compared to the Si-1 sample. This difference shows faster kinetics of Li_7_Si_12_ formation for the Si-2 sample. Since this reaction is related to delithiation of bulk silicon (core), it should be independent of the surface chemistry (e.g., oxide layer thickens and morphology). We believe that smaller size of the Si_EL_ core (for the Si-2 sample) resulted in a higher ratio of O1:O2 reaction rates due to lower internal stress generation. This lower stress magnitude originates from the good separation of the Si grains by the oxide matrix [7].

The average peak current of the Si-2 electrode is 4–6 times lower compared to the Si-1 sample. The overall reaction rate is much slower in the Si-2 electrode due to a large amount of poorly conductive silicon oxide which limits the electrical conductivity of the active mass. On the other hand, the large amount of silicon oxide can also easily limit the electrolyte access to the silicon core leading to mass transport limitations. Regardless of the limitation mechanism, the Si-2 electrode electrochemical resistance is much higher leading to smaller current density capabilities.

Different factors can affect the electrochemical results obtained for the silicon-based electrodes. The common problems observed for some Li-ion systems are poor electrical conductivity of the material or insufficient electrolyte penetration into the active mass. Those restrictions result in overpotential generation visible as peak flattering, shifting and broadening in CV experiments, as well as in poor capacity obtained for fast (high current) galvanostatic (GA) cycling. It should have marginal effect on the capacity obtained during slow GA cycling because the generated overpotential is directly proportional to the current density.

The third factor affecting the electrochemical results is distinctive for silicon (and few other alloy-type materials). It is related to internal pressure generation during lithium uptake (Si-Li alloy volume change). This pressure generates the overpotential in the range of 60–125 mV/GPa, while Si internal stress induced by lithiation can be as high as 1–3 GPa [16]. As the result, the lithiation reaction can be slowed down or completely stopped [14,17]. The stress-induced overpotential is affected by current density only in small extent and should result in a similar GA capacity drop and CV peak area reduction.

**Figure 6:**
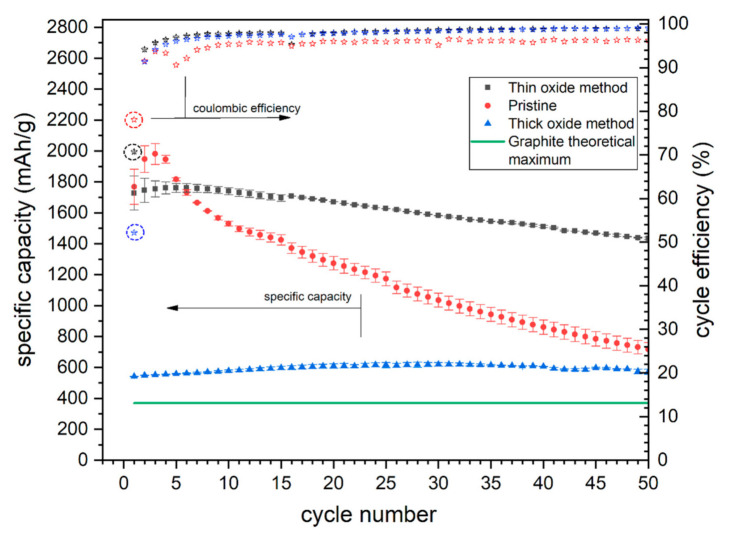
Pristine (red), Si-1 (black) and Si-2 (blue) galvanostatic charge/discharge results. Stars—coulombic efficiency. Squares, circles and triangles—specific delithiation capacities. Green line—theoretical capacity of graphite (372 mAh/g).

The galvanostatic cycling of the modified materials showed major performance differences (Figure 6). The initial specific capacity of the Si-1 sample (thin surface oxide) is almost the same as revealed by the pristine material (silicon NP’s). During a few subsequent cycles, the pristine material capacity increased by 12%, suggesting electrode structure changes resulting in an improvement of the electrolyte penetration into the active mass or smaller stress generation. The Si-1 electrode capacity remained almost constant between five initial cycles (increase of only 1.8%) suggesting an initial good electrolyte access to deeper electrode regions and fairly stable electrode macro-structure. After the 10th cycle both: pristine and Si-1 electrode presented close to linear capacity drop, although the capacity retention of the modified sample (Si-1) was much better and reached 83% after 50 cycles, in comparison with 40% for pristine silicon NP’s. The significant difference in the capacity retention between those two materials is the effect of diverse surface oxide characteristics. As indicated by XPS and CV experiments, the Si-1 powder is covered with a thin, but uniform and electrochemically resistant oxide layer. The thickness of the oxide is generally higher for the modified sample which results in a better volume changes buffering, as well as an improved SEI stability. The maximum cycle efficiency (Figure 6) increased from 96.2% (for pure silicon NP’s) to 99.1% after material modification. A much better SEI stability results in a less electrode degradation and a slower internal resistance buildup during cycling, thus can be assigned as one of the reasons for a better Si-1 capacity retention. The first cycle efficiency (star in a circle in Figure 6) is slightly lower for the modified sample (71% vs 78%) indicating that some amount of the oxide layer might be reduced during the first lithiation or the specific surface of the Si-1 sample increased after Si NPs modification. The first cycle Coulombic efficiency difference between the pristine and Si-1 modified sample is still relatively small, which can be attributed to the high electrochemical stability of the oxide layer at the modified silicon surface. Because of the high stability and a very high initial specific capacity, the Si-1 material can be directly used as an active material in Li-ion batteries.

The Si-2 electrode behavior during cycling was much different compared to both pristine and Si-1 modified material. The Si-2 electrode reveals an initial capacity close to 550 mAh/g and maintains almost a constant capacity during 50 cycles. The obtained capacity is almost 50% greater compared to graphite. A slight capacity increase occurred up to 30th cycle (14% gain). A comparison between CV and cycling data shows that the lithiation/delithiation reactions kinetic greatly increases during the first 20 cycles as indicated by an over 300% increase in the oxidation peak current in CV experiment, while the specific capacity change during these cycles are only minor. The Si-2 electrode reaction rates (and related peak currents) raise during cycling. This phenomenon is probably related to a couple of factors. One of them is an ongoing transformation of SiO_2_ (insulator) into Li_x_SiO_y_ (2.7 S/cm [42]). It can also originate from electrode structure changes induced by silicon volume variations. After several shrinking/expanding cycles, the silicon-based electrode structure usually becomes loose and a better electrolyte access into silicon grains is possible [11]. On the other hand, the loose electrode matrix structure leads to unrestricted silicon volume changes. The tight electrode structure of Si-2 sample may therefore limit the electrode capacity during a few initial cycles. After a couple of cycles the loosened structure generates less pressure on the expanding Si-grains and thus the overall capacity increases [7]. This demonstrates that a high compressive stress generated during the Si particle expansion can completely block the ongoing electrochemical reaction. Lack of oxidation peaks height increase for silicon NP’s and Si-1 materials (Figure S1 and Figure 4 respectively) is an additional indication of its intrinsic looser structure.

Generally, the stress induced lithiation restriction should affect both the galvanostatic cycling capacity and CV peak height. Since the galvanostatic cycling was performed using a small current density, the polarization caused by poor electric conductivity or electrolyte access limitations should have only a minor effect on the obtained capacity. The differences between GA capacity profiles during initial cycles indicates that stress induced lithiation restrictions (observed for the Si-1 sample) were much smaller in the Si-2 electrode, however it was not the only factor responsible for the different electrochemical response of these samples. If the large compressive stress were the only factor, the CV peak current and specific capacity gain in the initial cycles should present a similar magnitude. Thus, the relatively small capacity gain during cycling and the large oxidation peak current increase during CV of the Si-2 sample must originate from a better active layer penetration by the electrolyte. The smaller CV peak height changes for the Si-1 sample indicated that the electrolyte access was sufficient from the beginning of the 1st cycle. The very thick oxide layer formed at the Si-2 material resulted in a decrease of the electrolyte access during the initial cycles. The electrode structure changes induced by Si swelling/shrinking promotes better electrolyte penetration into the active mass after prolonged cycling.

The capacity of Si-2 electrode was almost constant during the first 50 cycles. The experiment was then continued up to 90 cycles (Figure S2). The electrode retained over 98% of initial capacity after this period. The great capacity retention proves that very thick oxide layer formed at the Si-02 powder during the modification procedure can provide an elastic matrix and thus greatly suppress the electrode degradation induced by silicon volume changes during cycling. Unfortunately, the first cycle efficiency of Si-2 electrode was very low (52%). The main reason for this effect is the severe reduction of the oxide as revealed by CV experiments. The poor initial cycle efficiency is a common problem observed for SiO_x_-based electrodes. Nevertheless, the efficiency value recorded for the Si-2 sample is higher compared to the reported in the literature for SiO_x_-based electrodes (30–48%) [43,44]. Furthermore the Si-2 material maintains a similar or higher specific capacity and capacity retention [43,44]. After the initial cycle, the efficiency quickly increases and stabilizes at 99.3% indicating very good electrode structure and SEI stability. Even though the initial oxide reduction is greatly limiting for the practical application of the modified Si-2 material, the use of the pre-lithiation step may greatly improve the material characteristics and applicability in Li-ion batteries. The pre-lithiation process should result in an improvement of several electrochemical parameters. First, after pre-lithiation the SEI forms naturally on the electrode surface due to its low initial potential. Secondly, the Si-oxides are reduced to Si and Li_2_O prior to the cell operation, leading to a great first cycle efficiency improvement (lower IRR) and a great reduction of the lithium losses. Several different pre-lithiation techniques had been proposed so far. A high-energy milling of silicon with lithium [45], a sacrificial lithium electrode utilization [46] or a stabilized lithium NPs (SLMP) addition into the active mass [47,48] were successfully developed at the lab-scale. Unfortunately, the utilization of these methods at the industrial scale is currently very difficult due to the high reactivity of pre-lithiated materials. Nevertheless, the utilization of pre-lithiation should result in first cycle IRR drop, and consequently, in successful application of Si-2 material.

## 4. Conclusions

In this work we presented two methods of silicon NP chemical oxidation. Both of these methods resulted in oxide formation at the surface of Si NPs which greatly altered their electrochemical characteristics. The combined XPS, EDS and SEM results proved that the oxide layers present significant differences among individual samples. The CV experiment revealed that the obtained surface oxides show a different susceptibility to electroreduction during the first lithiation. The one-step piranha solution modification resulted in a thin and very stable oxide coating that acts as an artificial SEI layer during electrode operation. The two-step modification with HF and piranha solution formed a very thick oxide layer that greatly suppressed the effects of silicon volume changes, but was prone to reduction. Both modification techniques are simple and scalable to the industrial level. The modified materials showed great prospects for application in commercial Li-ion batteries due to their high specific capacity and stability (capacity retention). While a thin oxide layer material can be directly implemented in the Li-ion system, the thick oxide layer material needs a further pre-lithiation prior to utilization in a real battery system. The presented modification methods are significantly beneficial for silicon-based Li-ion battery applications.

## Figures and Tables

**Table 1:** Surface atomic concentration of different elements/compounds found by XPS analysis.

	Pristine Powder	Si-1 (Modified)	Si-2 (Modified)
Elemental Si (at.%)	26.1	21.4	2.1
SiO (at.%)	2.6	3.2	
SiO_2_ (at.%)	71.3	75.5	97.9
F (all forms) (at.%)			2.1
S (SO_4_) (at.%)	10.0	11.5	5.5
N (all forms) (at.%)	2.7		1.3

**Table 2:** A relative content of silicon and oxygen content in the measured samples obtained by EDX. The oxide thickness calculated based on the r0 = 50 nm, Si density = 2.3 g/cm^3^, SiO_2_ density = 2.2 g/cm^3^.

Sample	Si (at.%)	O (at.%)	O/Si Ratio	Estimated Oxide Thickness (nm)
Pristine NPs	72.0	28.0	0.39	6.6
Si-01	67.7	32.3	0.48	8.2
Si-02	47.5	52.5	1.10	17.8

## Supplementary Materials

Figure S1: Pristine powder CV scan at 0.2 mV/; Figure S2: Specific capacity and cycle efficiency of Si-02 electrodes; Figure S3: The absolute composition of pristine and modified samples obtained by EDX; Figure S4: Pristine and modified samples composition. Silicon and oxygen relative content obtained by EDX.
