# Supplementary material for: Surface Oxidation of Nano-Silicon as a Method for Cycle Life Enhancement of Li-ion Active Materials
Source: Molecules. 2020 Sep 7;25(18):4093. doi: 10.3390/molecules25184093 (PMC7570913; doi:10.3390/molecules25184093)
Supplement: Supplementary file 1 [file molecules-25-04093-s001.pdf]

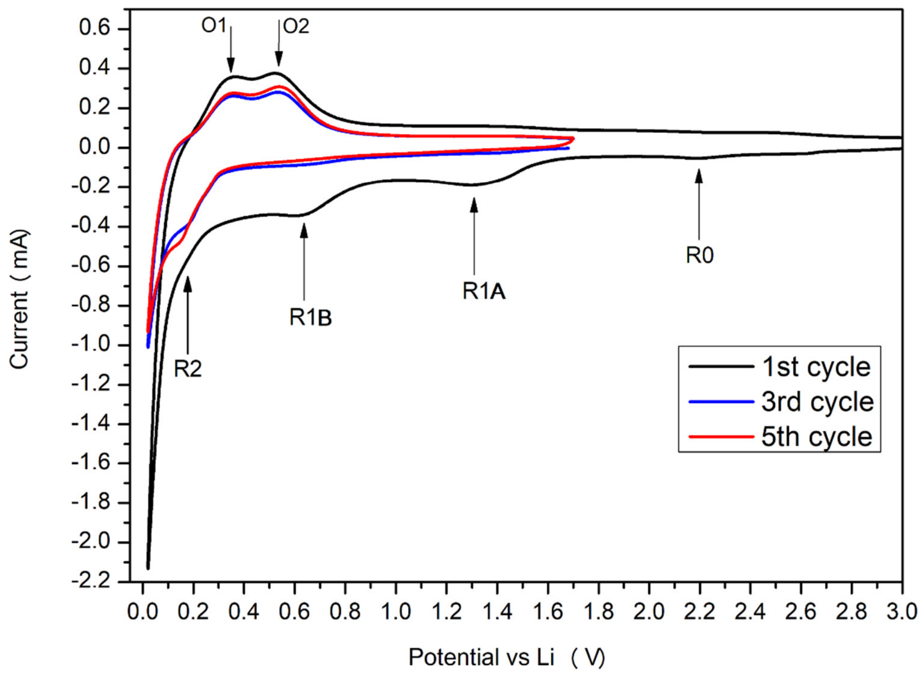

Figure S1. Pristine powder CV scan at 0.2 mV/s

The pristine sample CV scan revealed four different reduction peaks. The R0 and R1A peaks (Fig. S1) are attributed to the silicon oxides reduction leading to lithium silicates and lithium oxide formation. Those peaks diminish after the firs cycle. The R1B peak is directly related with the reduction of electrolyte components and SEI formation. The passivation layer was mostly formed during the first cycle, however very small R1B peak is still visible in the third CV scan indicating slow layer formation or initial instability. The R1B peak completely vanished in the subsequent cycles. The R2 peak arising in the third cycle is related to silicon lithiation mechanism change from a single kinetically limited reaction in the first cycle to the two separate reactions limited by the lithium diffusion. The cathodic part of the voltammetry profile recorded for the pristine sample is similar to the one observed during the Si-2 modified powder analysis. The important differences between those two are related to the much thicker oxide layer at the surface of Si-2 grains. The thicker oxide layer resulted in lower electrical conductivity causing R1A peak shift toward lower potential compared to pristine sample, and extreme broadening of the SEI formation peak. The SEI formation occurred mostly at 0.65 V for pristine sample, while the reaction at the Si-2 surface was retarded and occurred up to 0.2 V.

The anodic part of the CV scan presented great similarity between pristine material and Si-2 sample. The O1 and O2 height ratio was close to one. Their position was similar, however small shift towards higher potential is visible for pristine material. The shift value is about 50 mV and may originate from small alterations during sample preparation/cell assembly.

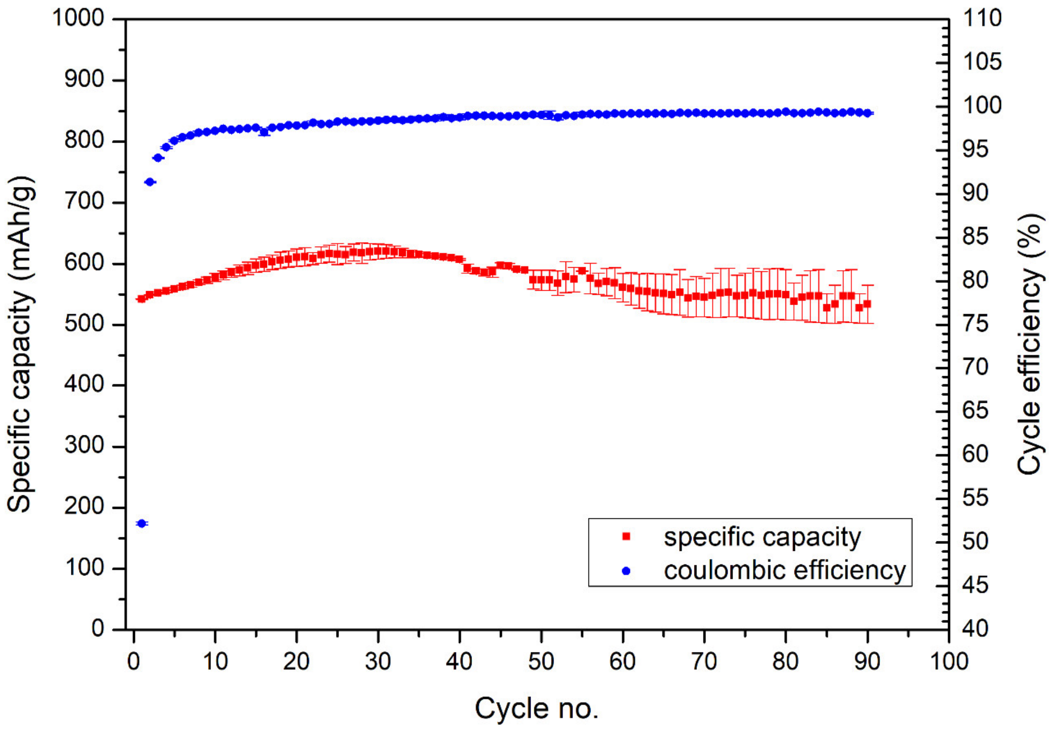

Figure S2. Specific capacity and cycle efficiency of Si-02 electrodes.

EDX analysis was performed at 15 kV accelerating voltage. The average excitation depth was 2.2 micrometer for all of measured samples. The average particle size (~100 nm), was much less that excitation depth, thus by the EDX analysis we obtained the average elemental composition of the whole particles volume (Fig. S3).

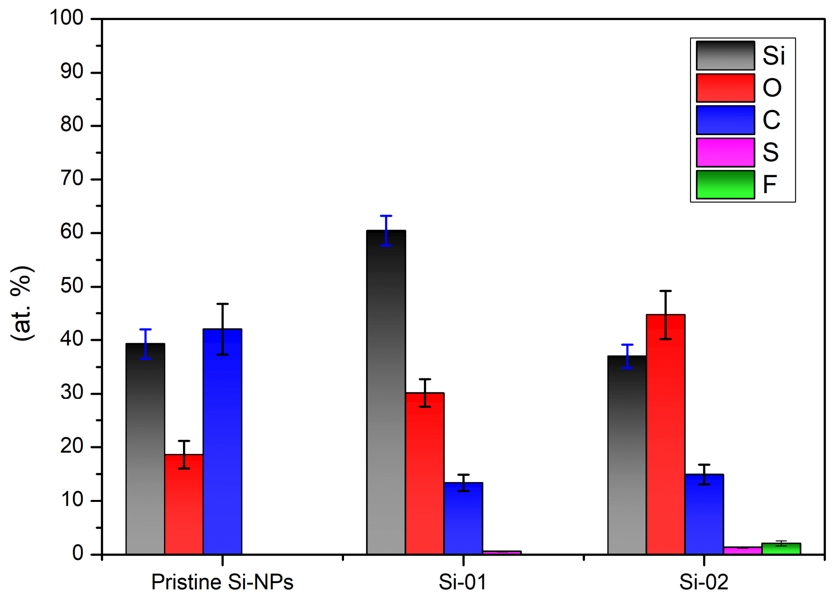

Figure S3. The absolute composition of pristine and modified samples obtained by EDX

Low amount of sulfur and fluorine contamination is related to the samples modification procedures as described in the main text. The high carbon content is mostly related to the signal from carbon-based adhesive foil that was used for sample immobilization during EDX measurement.

When the signal from carbon and contaminations (sulfur, fluorine) was excluded from the calculations we obtained the relative silicon and oxygen content in the samples. The Si-01 sample have the silicon and oxygen content very similar to the unmodified Si-NPs. Slight increase in the O/Si ratio (0.48) compared to the pristine sample (0.39) is in a good agreement with the XPS analysis. The Si-02 sample present much higher oxygen content. The O/Si ratio is over 1.10 indicating major oxidation of the Si-02 particles. The amount of elemental silicon in the measured samples is hard to determine, because the existence of SiO_x species (which is very probable) will affect the calculation precision. Regardless the accuracy The O/Si ratio is much lower than 2 (for pure SiO_2) indicating that the presence of elemental silicon under the surface oxide layer is expected.

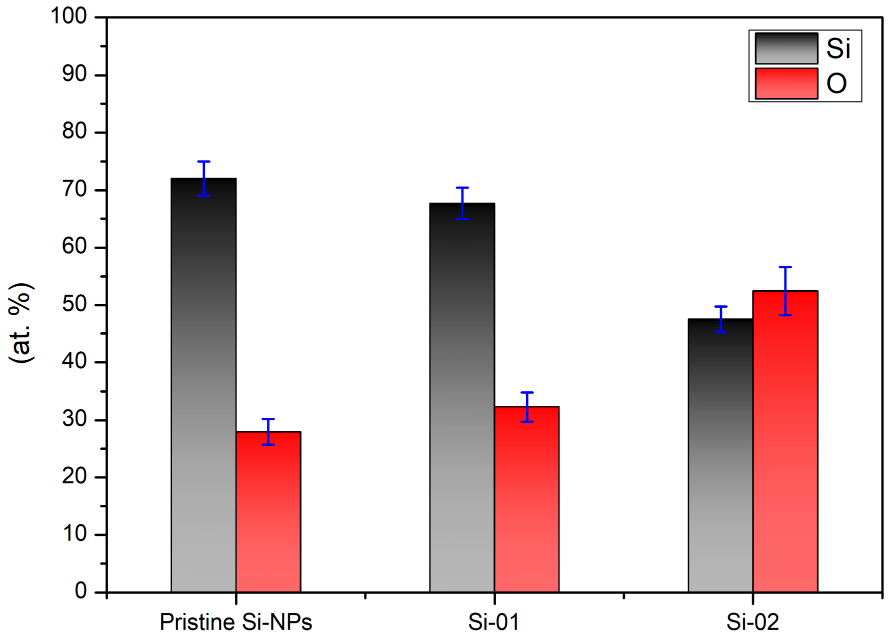

Figure S4. Pristine and modified samples composition. Silicon and oxygen relative content obtained by EDX
